# Intervention Mapping as a Guide for the Development of a Diabetes Peer Support Intervention in Rural Alabama

**DOI:** 10.5888/pcd9.110053

**Published:** 2012-01-12

**Authors:** Andrea Cherrington, Michelle Y. Martin, Michaela Hayes, Jewell H. Halanych, Susan J. Andreae, Monika Safford, Mary Annette Wright, Susan J. Appel

**Affiliations:** Division of Preventive Medicine, University of Alabama at Birmingham; School of Medicine, University of Alabama at Birmingham, Birmingham, Alabama; School of Medicine, University of Alabama at Birmingham, Birmingham, Alabama; School of Medicine, University of Alabama at Birmingham, Birmingham, Alabama; School of Medicine, University of Alabama at Birmingham, Birmingham, Alabama; School of Medicine, University of Alabama at Birmingham, Birmingham, Alabama; School of Nursing, University of Alabama at Birmingham, Birmingham, Alabama; School of Nursing, University of Alabama at Birmingham, Birmingham, Alabama

## Abstract

**Introduction:**

Peer support is a promising strategy for the reduction of diabetes-related health disparities; however, few studies describe the development of such strategies in enough detail to allow for replication. The objective of this article is to describe the development of a 1-year peer support intervention to improve diabetes self-management among African American adults with diabetes in Alabama's Black Belt.

**Methods:**

We used principles of intervention mapping, including literature review, interviews with key informants, and a discussion group with community health workers, to guide intervention development. Qualitative data were combined with behavioral constructs and principles of diabetes self-management to create a peer support intervention to be delivered by trained peer advisors. Feedback from a 1-month pilot was used to modify the training and intervention.

**Results:**

The resulting intervention includes a 2-day training for peer advisors, who were each paired with 3 to 6 clients. A one-on-one in-person needs assessment begins an intensive intervention phase conducted via telephone for 8 to 12 weeks, followed by a maintenance phase of at least once monthly contacts for the remainder of the intervention period. A peer support network and process measures collected monthly throughout the study supplement formal data collection points at baseline, 6 months, and 12 months.

**Discussion:**

Intervention mapping provided a useful framework for the development of culturally relevant diabetes peer support intervention for African Americans living in Alabama's Black Belt. The process described could be implemented by others in public health to develop or adapt programs suitable for their particular community or context.

## Introduction

The prevalence of type 2 diabetes is increasing (1). In the United States, the disease disproportionately affects minority populations, particularly African Americans in the rural South (2). Proper diabetes self-management can lead to improved glycemic control, blood pressure, and lipid levels, and can mitigate the negative health effects of comorbid conditions (3,4). However, the demands of self-management behaviors (eg, diet modification, physical activity, medication adherence, self-monitoring of blood glucose levels) can be difficult to balance and are influenced by socioeconomic, cultural, and psychosocial factors such as lack of social support, self-efficacy, coping skills, and increased barriers to self-care (2,5,6). Residents of rural areas face additional barriers to diabetes self-management, including limited access to health care services, providers, and education programs; high rates of poverty; low levels of health literacy; and increased distances from social networks (7).

These conditions are widespread in Alabama's Black Belt region. Named for its dark soil and agricultural history, the Black Belt encompasses approximately 18 counties in southern Alabama. African Americans comprise more than 30% of the population; rates are as high as 80% in some areas. In this region, more than 30% of African Americans over the age of 50 have diagnosed diabetes.

As diabetes self-management has received more attention, strategies (eg, increasing social support) have been proposed to meet the needs of those living with diabetes (5,8). However, such programs may be difficult to implement in rural settings, and limited effects of classic support (ie, family/spouse) have strengthened the case for peer-involved programs (7,8).

Peer advisors are ideal for community-based support programs because of their ability to serve in a reciprocal, nonhierarchical capacity. In general, peer advisors are nonprofessionals with an intimate knowledge of the difficulties of disease management who can provide support on the basis of shared life experiences (9,10). Peer-based programs improve health behaviors as well as health status (5). For example, a recent peer advisor program demonstrated improved communication with health care providers, use of community resources, and diabetes management (11). Studies in the United States indicate that peer advisors may be successful in minority populations and with people who may distrust traditional health care systems (12,13).

In rural settings, the combination of peer advisors with telephone-based support may be particularly effective; it eliminates barriers such as transportation, costs of group attendance, and time constraints (7,14). Studies of telephone interventions show that they help improve glycemic control (2,15).

The objective of this study is to describe our experience with the use of intervention mapping as a guide for the development of a peer support diabetes intervention for a rural, medically underserved population (16). Intervention mapping is a systematic process that combines theory, empirical evidence from the literature, and data from the community to develop health education programs. The process involves multiple steps that begin with a community assessment and continues by fostering collaborations with community stakeholders during intervention development and program planning. Intervention mapping has been used successfully to develop health behavior programs, mostly related to cancer (17,18).

## Methods

Intervention mapping is an iterative process encompassing 6 key stages. For several years before the grant funding for the peer support intervention, we conducted the literature review and semi-structured interviews. This preliminary work provided investigators with a foundation from which to respond to the Peers for Progress call for proposals; we worked with existing community partners to develop the study design. After funding was received in 2009, 8 months were spent developing the intervention, which included conducting unstructured interviews with community members and a discussion group with community members of an existing community health worker (CHW) network.

### Step 1. Needs assessment

The needs assessment was based on 1) a literature review of existing diabetes programs involving peers or lay health workers, 2) nationwide field experience, and 3) local needs assessment.

**Figure 1.**
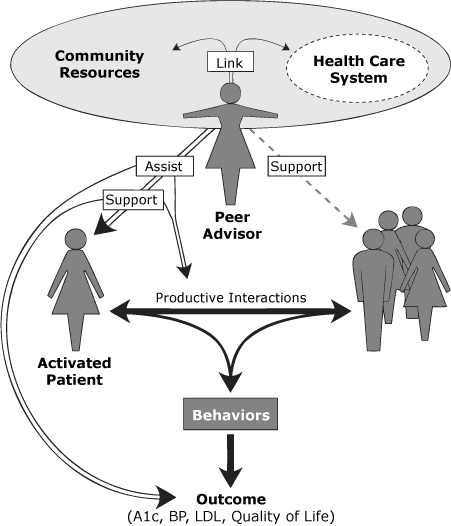
Role of peer advisors in diabetes management — assist, support, and link, in the context of the chronic care model. Abbreviations: A1c, hemoglobin A1c; BP, blood pressure; LDL, low-density lipoprotein cholesterol.

1. Literature review. A review of diabetes programs involving peer advisors around the United States was conducted, the results of which have been published elsewhere (9). Briefly, we found that peer advisor roles, responsibilities, and training varied across programs and were context-specific. After our review, the World Health Organization (WHO) identified 3 main peer advisor roles related to diabetes self-management: to link, to assist, and to support (19). We used information from our own review and from the WHO report to conceptualize the peer advisor role (Figure 1).

2. Field experience. In parallel with our literature review, semi-structured interviews were conducted with program managers from diabetes peer advisor programs across the country, allowing for an in-depth exploration of barriers and facilitators to implementation. This work indicated that peer advisors are prone to burnout and the positions are prone to turnover; remuneration, both monetary and nonmonetary incentives, is another consideration. A full report of the results has been published elsewhere (20). These lessons influenced the intervention structure and content. Specifically, plans were made for a manageable caseload (3-6) with limited paperwork and simplified forms. Additionally, support networks were designed to facilitate interaction between peers and extend to investigators and community coordinators.

3. Local needs assessment. The priority population for this study was African American adults with diabetes living in Alabama's Black Belt. Assessments conducted by the Alabama Department of Public Health (ADPH) have documented racial disparities related to diabetes and associated complications, particularly in many Black Belt counties. ADPH determined that, when stratified by race and sex, diabetes prevalence among African American women was 7% higher than among white women (21). Furthermore, diabetes prevalence was significantly higher in Alabama residents whose annual household incomes were less than $35,000 (21). Many of these areas also lack primary health care providers, endocrinologists, and other resources, including diabetes education. ADPH reported in 2010 that endocrinologists and diabetes educators in Alabama are located in mostly urban counties with higher populations (21). For example, 57 of the 67 counties have no endocrinologists, and 45 of 67 counties have no certified diabetes educators (21). Socioeconomic constraints further exacerbate the problem; one-third of Black Belt residents have incomes below the federal poverty level (22). Informal needs assessments conducted among members of existing cancer community coalitions revealed that diabetes was an area of concern among community members and that a program focused on diabetes was a natural next step. Furthermore, discussions with community members and staff at partnering practices revealed that a formal diabetes education program would benefit the community. Thus, in the design phases of the study, it was decided that both trial arms would include diabetes education.

### Step 2: Identifying outcomes and change objectives

**Figure 2.**
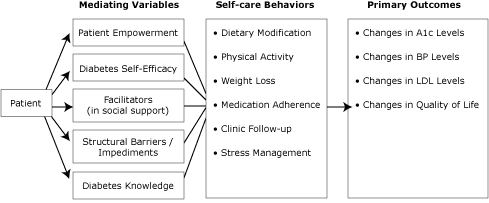
Development of peer support intervention: mediating variables, self-care behaviors, and primary outcomes. Abbreviations: A1c, hemoglobin A1c; BP, blood pressure; LDL, low density lipoprotein.

Results from Step 1 were used to identify outcomes and change objectives. Although the overarching objective of this intervention is to improve glycemic control, blood pressure, and lipid levels through a peer advisor intervention designed to improve self-care, proximal measures, including self-care behaviors, were selected as secondary outcomes (Figure 2). Additionally, we included patient-centered outcomes such as quality of life, depressive symptoms, and stress. Similarly, we were interested in potential antecedents to or mediators of those behaviors, such as self-efficacy and patient empowerment. These complemented our theoretical basis and our plans for peer support.

**Table:** 

	**Medicating Variables**	**Self-Care Behaviors**	**Primary Outcomes**
**Patient**	Patient empowerment Diabetes self-efficacy Facilitators (in social support) Structural barriers/impediments Diabetes knowledge	Dietary modification Physical activity Weight loss Medication adherence Clinic follow-up Stress management	Changes in A1c levels Changes in BP levels Changes in LDL levels Changes in quality of life

### Step 3: Selecting theory-based methods and practical strategies

The chronic care model served as a conceptual framework for the overall intervention (23). In previous work, we have proposed integrating peer advisors into the chronic care model at the intersection of community and health care organizations (20). For this study, we conceptualized the peer advisors as conducting activities within the community, helping participants access resources within the community and the health care system. The theoretical foundation of our intervention combined several complementary theories, including the health belief model, social cognitive theory, adult learning theory and empowerment model, and social networks and social support, each of which has been applied to diabetes management in previous studies (Appendix A) (13,24-28). Grounding the intervention in behavior change theory combined with measures tied to theory-based constructs allowed us to understand the pathways to any intervention effects.

Practical strategies for intervention delivery were based on evaluation of literature and existing peer-based programs (described earlier), including an assessment of specific needs of volunteers (29). Strategies were further informed by a discussion group with 7 peer advisors from previous cancer awareness projects in the Black Belt. The purpose of the discussion group was to solicit feedback on the structure of the peer advisor program, recruitment and retention strategies, methods for intervention delivery, and perceived needs. Additional input was solicited for developing training manuals, educational materials, and participant incentives.

### Step 4: Developing the program

Because many communities in rural Alabama lack access to diabetes education, we chose to develop a diabetes education program for both intervention and control participants. For the peer support intervention, we hypothesized that people with diabetes coached by trained peer advisors will experience more observational learning opportunities, reinforcement, and social support, leading to improved health behaviors, metabolic measurements, and, ultimately, quality of life. On the basis of a literature review, the team felt that people who either lived with diabetes themselves or helped a close friend or family member with their daily diabetes self-care would be the best peer advisors (29).

We then conducted a 4-week pilot of the training and the intervention. We trained 2 peer advisors and recruited 7 participants from a community health center, from an existing community diabetes support group, and by word of mouth. Peer advisors met their clients on enrollment day to conduct the needs assessment and then called their clients weekly. Peer advisors also had weekly calls with investigators to report on progress and troubleshoot problems. The calls provided an opportunity to observe intervention fidelity, reinforce skills taught during training, and offer support.

## Results

The education program consists of three 30-minute interactive learning modules led by nurses or trained health educators. Content is based on information from the American Diabetes Association's 2009 Standards of Care Guidelines (30) and from a segment we developed that focuses on "raising the BAR (Be prepared, Ask and learn, Reflect and reach out) on your doctor visit." The learning modules include 1) diabetes basics and the office visit, 2) healthy eating, and 3) exercise and stress management (Appendix B).

We planned for peer advisors to provide a flexible, informal approach to assisting patients, based on role modeling and patient empowerment/activation, rather than formal delivery of a diabetes education curriculum. We identified and developed training activities consistent with 3 main roles: assist, support, and link (Figure 1). Training content included discussion of peer advisor roles and expectations, principles of motivational interviewing and communication, goal-setting and problem-solving, human subjects in research protections, health care and community resources, diabetes basics, and training on the protocol and forms required for process data collection.

The intervention begins with the peer advisor conducting an in-person needs assessment to identify an area of diabetes management that the client wants to improve. The needs assessment reviews medication adherence, diet, exercise, stress, and talking to their doctor. Using motivational interviewing techniques, the peer advisor guides the participant to set 1 SMART (specific, measurable, achievable, realistic, time-oriented) goal.

After the initial meeting, the peer advisor contacts the client weekly by telephone for 8 to 12 weeks (intensive intervention phase) and at least monthly thereafter (maintenance phase). At each telephone contact, the peer advisor asks the client about new problems, reviews the client's SMART goal, and if the goal has been met, collaboratively sets a new goal. If the client does not want to discuss the goal, the peer advisor provides support and documents the discussion. If the client is having trouble attaining the goal, the peer assists the client in identifying barriers and problem-solving.

In a separate contact made a day or 2 before an office visit, the peer and client plan for the visit and develop a strategy for asking questions and understanding recommendations. Another contact is made a day or 2 after the visit to review what occurred, make plans as needed, and, if necessary, recontact the office to address unmet needs.

### Pilot testing the intervention

We conducted a 4-week pilot of the training and the intervention. After completion of the pilot, study investigators conducted two 1-hour group interviews, one with peer advisors and one with the clients. Overall, the intervention was well received and the study forms were deemed reasonable. Both peers and clients felt that transitioning to monthly calls after 8 weeks may not provide enough support and recommended a flexible, client-tailored approach to lengthening the call interval. The intervention was modified accordingly.

Feedback from the pilot peer advisors also led us to create a peer support network. Peers assigned to a particular region were asked to function as a team to enhance retention and morale, minimize burnout, and permit case reassignment should a peer drop out. Monthly support meetings were planned during the intervention period, and each peer advisor was paired with another peer advisor from their area as a one-on-one supporter.

### Steps 5 and 6: Planning for implementation and evaluation

To aid implementation and adoption, information from the pilot was used to modify the peer training manual so that it could serve both as a guide for the training sessions and as an ongoing resource during the intervention period and beyond. Currently, the program is being tested in a group-randomized trial. More than 60 peer advisors have completed training, 424 participants have been enrolled, and 200 have been matched to peer advisors.

Evaluation measures include biometric measures (hemoglobin A1c, blood pressure, low-density lipoprotein cholesterol, body mass index, and waist circumference) and patient-centered measures along with theory-based behavioral outcomes (see step 2). Measures have been collected at baseline and will be collected again 6 and 12 months later. To facilitate this process, the study team trained several community members to assist with data collection, including biometric assessments and face-to-face interviews.

Process measures are being collected from peer advisors. Using the pilot-tested contact forms, peers document each contact, both scheduled and spontaneous, and, through separate forms, contacts before and after the office visit. Forms are submitted to community coordinators at monthly meetings and evaluated by coordinators and the investigative team for prompt action should deficits be identified. In addition, telephone contacts between investigators, groups of 3 to 4 peer advisors, and the community coordinator take place weekly for the first 4 weeks and at 6-week intervals during the maintenance phase. These calls allow investigators and peers to review progress, troubleshoot problems, and reinforce training.

## Discussion

The systematic and collaborative approach to program development described in this article is guided by principles of intervention mapping in the context of community-based participatory research. The 6-step intervention mapping framework resulted in a peer-delivered diabetes self-management program that was informed by health behavior theory and local context to be responsive to community needs. The resultant intervention is patient-centered, peer-delivered via telephone, and focuses on building goal-setting and problem-solving skills. Community involvement in the early planning stages led to adjustments in program design and implementation that, although specific to this study, may have implications for programs targeting rural areas more broadly.

For most communities, willingness to partner with research institutions is balanced with the desire to advocate for needed resources. Randomized controlled trials are often viewed with skepticism as individuals or entire communities risk being relegated to the control arm, often without tangible benefits (31-33). The need to respond to community-identified needs and create sustainable partnerships becomes more apparent as research efforts increasingly focus on translating interventions into real-world settings. This is evident in the increasing number of investigators employing community-based participatory research methods.

Although implementing alternative designs to randomized controlled trials can overcome the resistance by many communities to the control arm, our development process, particularly feedback obtained from partnering practices and community leaders, provided us with a potential solution. We elected to develop education services for both trial arms. In the Black Belt, community health centers and organizations attempt to provide quality health care with very limited resources, and formal diabetes education is unavailable. In this study, early discussions with providers and key community leaders even before applying for funding confirmed the importance of providing education to all participants, and our community partners cited the offer of such a program as a key component of successful recruitment efforts and community engagement.

Early formative work with community partners also proved essential for developing support strategies for peer counselors. Previous studies have found that peer advisors are prone to burnout, leading to high turnover rates (20). Contributing factors include perceived isolation and lack of ongoing support, coupled with a high caseload and heavy paperwork (20,34-36). These lessons were confirmed by discussions with existing peer advisors in the Black Belt. Therefore, plans were made during the pilot phase for weekly calls between investigators and peers to debrief and provide support. A "buddy system" and a regional support group added another dimension of peer support. The challenge of this extensive approach is the need for significant investigator and study staff time and resources. However, investigators profited considerably from these labor-intensive contacts, obtaining reassurance on intervention fidelity, having opportunities to reinforce training, and deepening bonds with the peer advisors. Future studies are needed to better understand peer advisor support needs and to develop sustainable strategies to deliver such support beyond the life of  study.

Intervention mapping was a comprehensive approach to community-based intervention development that was both highly regarded by our community partners and scientifically rigorous. Key features included engaging community members from the beginning, even before applying for grant funding, and staying engaged throughout program development and implementation. Although details and specific procedures were designed for and with African Americans living in Alabama's Black Belt and may not be generalizable to other settings, our experience and strategies may be helpful for others developing interventions in underserved communities.

## Appendices

### Appendix A. Diabetes Peer Intervention: Behavioral Theories, Peer Advisor Activities, and Outcome Measures

**Table:**

Intervention Goal	Underlying Theory	Constructs	Operational Definition	Peer Activities	Outcome Measure
Improve self-efficacy for self-management behaviors	Social cognitive theory	Self-efficacy and health behaviors	Increase self-efficacy for self-management (medication adherence, physical activity, increased fruit and vegetable intake, improved portion control) Self-efficacy for managing stress	Role model behaviors Assist with setting SMART goals Use nondirective skills to encourage empowerment	Medication adherence (37-39) Physical activity Fruit and vegetable intake, portion control^a^
Improve patient activation	Empowerment model	Patient activation	Belief that the patient role is important Have confidence to take action Have knowledge for improving one's health Staying the course under stress	Assist with goal setting, focusing on doctor visit Use nondirective skills to encourage empowerment	Patient Activation Measure (40) Perceived Efficacy in Patient-Physician Interactions (5 item) (41)
Decrease perceived barriers	Health belief model	Perceived barriers	Address barriers identified by patient	Teach problem solving skills	Adapted from Rodriguez et al, community health advisors assessment survey (42)
Increase perceived support	Social support theory, social cognitive theory, and health belief model	Social support: instrumental, emotional, and informational	Help patient identify/connect with community resources (instrumental) Provide social support (emotional) Provide diabetes information (informational)	Link patient to community resources Supportive listening Link to diabetes information	Social support survey (43)

Abbreviations: SMART, Specific, Measurable, Achievable, Realistic, Time.

a Combination of the Summary of Diabetes Self Care Activities Scale (SDSCA), modifications by Toobert, Fisher, and Glasgow. The SDSCA is a well-validated and comprehensive measure of self-care behaviors in diabetes. It has been revised recently and includes supplemental items in an expanded version for more detailed information on the specific activities.

### Appendix B. Components of Diabetes Education Program for Rural Patients With Diabetes (Intervention and Control Patients)

**Table:**

Diabetes Education	Station Content	Props and Handouts
Diabetes basics and "Raising the BAR on Your Doctor'sVisit"	This station focused on increasing the participant's knowledge of diabetes, diabetes control, and the importance of doctor's visits.Specific emphasis was placed on the following: Types of diabetes, diabetes risk factors. Diagnostic test and values. Medications, foot care, risk factors for diabetes complications. The ABCs (A1c, Blood Pressure, Cholesterol) of diabetes management. Some common myths about diabetes. Getting the most out of every doctor's visit, including Being prepared, Asking and learning, and Reflecting and reaching out.	Visual aids used to provoke discussion: Diabetes report card. Monofilament. Foot pictures showing cracked heels, corns, calluses. Handouts: "The ABCs of Diabetes Management" handout. "Raising the BAR on Your Doctor's Visit" handout. Pillbox. Mini notebook for use before and at the doctors' office.
Healthy eating	This station focused on increasing the participant's knowledge of healthy eating.Specific emphasis was placed on the following themes: Portion size. increasing the quantity of fresh fruits and vegetables while decreasing fats and starches. Foods that should be limited, foods that should be eaten in moderation, foods that may be eaten with few limitations were identified on the basis of the Traffic Light Diet. Making healthy fast-food and restaurant choices. Facts about sugar-sweetened drinks.	Visual aids used to provoke discussion: American Dietetic Association dietary portion sizes of artificial foods. 9-inch plate-method placemat. *Simply Good Cooking* cookbook from Uniontown, Alabama. Handouts: Healthy Eating handout. 9-inch plate-method placemat. Copy of *Simply Good Cooking*.
Exercise and stress management	This station focused on increasing the participant's knowledge of stress management, depression, and the importance of exercise.Focal points: Identifying and discussing coping techniques for stress management. Common stressors. Signs and symptoms of depression, and difference in depression and stress. Participation in a brief relaxation exercise. Also stressed was the importance of exercise for diabetes in general and as a stress-relieving strategy. Chair exercises were taught, and home exercise options were discussed during this educational station.	Visual aids used to provoke discussion: Stress ball. Chair exercises. Group relaxation activities. Handouts: "Stress Management/Depression" handout "Exercise" handout Stress ball Exercise DVD ("Walking Down Your Blood Sugar")

## References

[1] Wild S, Roglic G, Green A, Sicree R, King H (2004). Global prevalence of diabetes: estimates for the year 2000 and projections for 2030. Diabetes Care.

[2] Samuel-Hodge CD, Keyserling TC, France R, Ingram AF, Johnston LF, Davis LP (2006). A church-based diabetes self-management education program for African Americans with type 2 diabetes. Prev Chronic Dis.

[3] (1993). The effect of intensive treatment of diabetes on the development and progression of long-term complications in insulin-dependent diabetes mellitus. The Diabetes Control and Complications Trial Research Group. N Engl J Med.

[4] (1998). Intensive blood-glucose control with sulphonylureas or insulin compared with conventional treatment and risk of complications in patients with type 2 diabetes (UKPDS 33). UK Prospective Diabetes Study (UKPDS) Group. Lancet.

[5] Heisler M (2010). Different models to mobilize peer support to improve diabetes self-management and clinical outcomes: evidence, logistics, evaluation considerations and needs for future research. Fam Pract.

[6] Weijman I, Ros WJ, Rutten GE, Schaufeli WB, Schabracq MJ, Winnubst JA (2005). The role of work-related and personal factors in diabetes self-management. Patient Educ Couns.

[7] Massey CN, Appel SJ, Buchanan KL, Cherrington AL (2010). Improving diabetes care in rural communities: an overview of current initiatives and a call for renewed efforts. Clin Diabetes.

[8] van Dam HA, van der Horst FG, Knoops L, Ryckman RM, Crebolder HF, van den Borne BH (2005). Social support in diabetes: a systematic review of controlled intervention studies. Patient Educ Couns.

[9] Cherrington A, Ayala GX, Amick H, Scarinci I, Allison J, Corbie-Smith G (2008). Applying the community health worker model to diabetes management: using mixed methods to assess implementation and effectiveness. J Health Care Poor Underserved.

[10] Norris SL, Chowdhury FM, Van Le K, Horsley T, Brownstein JN, Zhang X (2006). Effectiveness of community health workers in the care of persons with diabetes. Diabet Med.

[11] Klug C, Toobert DJ, Fogerty M (2008). Healthy Changes for living with diabetes: an evidence-based community diabetes self-management program. Diabetes Educ.

[12] Eng E, Parker E, Harlan C (1997). Lay health advisor intervention strategies: a continuum from natural helping to paraprofessional helping. Health Educ Behav.

[13] Israel BA (1985). Social networks and social support: implications for natural helper and community level interventions. Health Educ Q.

[14] Ellis I (2004). Is telehealth the right tool for remote communities? Improving health status in rural Australia. Contemp Nurse.

[15] Tudiver F, Wolff LT, Morin PC, Teresi J, Palmas W, Starren J (2007). Primary care providers' perceptions of home diabetes telemedicine care in the IDEATel project. J Rural Health.

[16] Bartholomew LK, Parcel GS, Kok G, Gottlieb NH (2001). Intervention mapping: designing theory and evidence-based health promotion programs.

[17] McEachan RR, Lawton RJ, Jackson C, Conner M, Lunt J (2008). Evidence, theory and context: using intervention mapping to develop a worksite physical activity intervention. BMC Public Health.

[18] Scarinci IC, Bandura L, Hidalgo B, Cherrington A Development of a theory-based (PEN-3 and health belief model), culturally relevant intervention on cervical cancer prevention among Latina immigrants using intervention mapping. Health Promot Pract.

[19] Diabetes action online.

[20] Cherrington A, Ayala GX, Amick H, Allison J, Corbie-Smith G, Scarinci I (2008). Implementing the community health worker model within diabetes management: challenges and lessons learned from programs across the United States. Diabetes Educ.

[21] (2010). Diabetes in Alabama: a report from the Alabama Department of Public Health.

[22] US Census Bureau, Small Area Estimate Branch, Table 1: 2010 Poverty and Median Income Estimates, Counties.

[23] Wagner EH, Austin BT, Von Korff (1996). Organizing care for patients with chronic illness. Milbank Q.

[24] Bandura A (1986). Social foundations of thought and action: a social cognitive theory.

[25] Freire P (1970). Pedagogy of the oppressed.

[26] Israel BA (1982). Social networks and health status: linking theory, research, and practice. Patient Couns Health Educ.

[27] Janz NK, Becker MH (1984). The Health Belief Model: a decade later. Health Educ Q.

[28] Knowles MS (1990). The adult learner: a neglected species.

[29] Cherrington A, Ayala GX, Elder JP, Arredondo EM, Fouad M, Scarinci I (2010). Recognizing the diverse roles of community health workers in the elimination of health disparities: from paid staff to volunteers. Ethn Dis.

[30] American Diabetes Association (2009). Standards of medical care in diabetes — 2009. Diabetes Care.

[31] Thompson JR, Horton C, Flores C (2007). Advancing diabetes self-management in the Mexican American population: a community health worker model in a primary care setting. Diabetes Educ.

[32] Osrin D, Azad K, Fernandez A, Manandhar DS, Mwansambo CW, Tripathy P, Costello AM (2009). Ethical challenges in cluster randomized controlled trials: experiences from public health interventions in Africa and Asia. Bull World Health Organ.

[33] Adamson J, Cockayne S, Puffer S, Torgerson DJ (2006). Review of randomised trials using the post-randomised consent (Zelen's) design. Contemp Clin Trials.

[34] Freedman B (1987). Equipoise and the ethics of clinical research. N Engl J Med.

[35] Brown C, Hennings J, Caress AL, Partridge MR (2007). Lay educators in asthma self management: reflections on their training and experiences. Patient Educ Couns.

[36] Cherrington AL,  Agne A, Munoz M, Scarinci I (2011). Challenges faced by community health workers engaged in programs for Latino immigrants in the Southeastern United States [abstract]. Ann Behav Med.

[37] Gates LB, Akabas SH (2007). Developing strategies to integrate peer providers into the staff of mental health agencies. Adm Policy Ment Health.

[38] Morisky DE, Ang A, Krousel-Wood M, Ward HJ (2008). Predictive validity of a medication adherence measure in an outpatient setting. J Clin Hypertens (Greenwich).

[39] Krapek K, King K, Warren SS, George KG, Caputo DA, Mihelich K (2004). Medication adherence and associated hemoglobin A1c in type 2 diabetes. Ann Pharmacother.

[40] Hibbard JH, Stockard J, Mahoney ER, Tusler M (2004). Development of the Patient Activation Measure (PAM): conceptualizing and measuring activation in patients and consumers. Health Serv Res.

[41] Maly RC, Frank JC, Marshall GN, DiMatteo MR, Reuben DB (1998). Perceived efficacy in patient-physician interactions (PEPPI): validation of an instrument in older persons. J Am Geriatr Soc.

[42] Rodriguez VM, Conway TL, Woodruff SI, Edwards CC (2003). Pilot test of an assessment instrument for Latina community health advisors conducting an ETS intervention. J Immigr Health.

[43] Rosland AM, Kieffer E, Israel B, Cofield M, Palmisano G, Sinco B (2008). When is social support important? The association of family support and professional support with specific diabetes self-management behaviors. J Gen Intern Med.

